# Clinical and Technical Validation of OncoIndx^®^ Assay—A Comprehensive Genome Profiling Assay for Pan-Cancer Investigations

**DOI:** 10.3390/cancers16193415

**Published:** 2024-10-08

**Authors:** Aarthi Ramesh, Atul Bharde, Alain D’Souza, Bhagwat Jadhav, Sangeeta Prajapati, Kanchan Hariramani, Madhura Basavalingegowda, Sandhya Iyer, Sumit Halder, Mahesh Deochake, Hrishita Kothavade, Aravindan Vasudevan, Mohan Uttarwar, Jayant Khandare, Gowhar Shafi

**Affiliations:** 1 OneCell Diagnostics, 209 B, GO Square, Aundh-Hinjewadi Road Wakad, Pune 411057, Maharashtra, Indiaalain@onecelldx.com (A.D.); bhagwat@onecelldx.com (B.J.); mahesh.deochake@onecelldx.com (M.D.); aravindan@onecelldx.com (A.V.); mohan@onecelldx.com (M.U.); 2 OneCell Diagnostics, 20380 Town Center Lane, #218, Cupertino, CA 95014, USA; 3 OneCell Dx., Inc., Molecular Medicine Research Institute, 428, Oakmead Parkway, Sunnyvale, CA 94085, USA

**Keywords:** comprehensive genomic profiling (CGP), next-generation sequencing (NGS), artificial intelligence, cancer genomics, targeted gene sequencing

## Abstract

**Simple Summary:**

The OncoIndx^®^ platform is an NGS assay designed and developed to identify critical mutations that help in therapeutic decision-making. The OncoIndx^®^ panel targets major exons and a few selected introns of 1080 cancer-associated and actionable genes. The test analyzes complex biomarkers such as single nucleotide variants (SNVs), copy number alterations (CNAs), specific gene fusions, and many more. This study validates the overall sensitivity and efficiency of the test using standard references, clinical samples, and U.S. Food and Drug Administration (FDA)-approved cross-laboratory samples. The ultimate goal of this research is to benchmark the assay against the current guidelines and increase the reliability of the test, thus increasing the confidence of medical professionals for better personalized therapeutic intervention.

**Abstract:**

Comprehensive next-generation sequencing (NGS) assays enable the identification of clinically relevant mutations, enhancing the capability for targeted therapeutic interventions. In addition, genomic alterations driving the oncogenic roadmap and leading to resistance mechanisms are reshaping precision oncology. We report the workflow and clinical and technical validation of the OncoIndx^®^ NGS platform—a comprehensive genomic profiling (CGP)-based assay for pan-cancer investigation. We evaluated the concordance between the OncoIndx^®^ test findings and clinically established hotspot detection using SeraSeq reference standards. OncoIndx is a hybridization capture-based NGS assay for the targeted deep sequencing of all exons and selected introns of 1080 cancer-related genes. We show the outcome in the form of tier I and tier II single nucleotide variants (SNVs), copy number alterations (CNAs), and specific gene fusions. OncoIndx^®^ also informs genome-wide tumor mutational burden (TMB), microsatellite instability (MSI), homologous recombination deficiency (HRD), and genomic loss of heterozygosity (gLOH). A total of 63 samples were utilized for validation with reference standards, clinical samples, and orthogonal assessment for genomic alterations. In addition, 49 cross-laboratory samples were validated for microsatellite instability (MSI), and for the tumor mutation burden (TMB), 18 samples as reference standards, 6 cross-laboratory samples, and 29 TCGA samples were utilized. We show a maximum clinical sensitivity of 98% and a positive predictive value (PPV) of 100% for the clinically actionable genomic variants detected by the assay. In addition, we demonstrate analytical validation with the performance of the assay, limit of detection (LoD), precision, and orthogonal concordance for various types of SVs, CNAs, genomic rearrangements, and complex biomarkers like TMB, MSI, and HRD. The assay offers reliable genomic predictions with the high-precision detection of actionable variants, validated by established reference standards.

## 1. Introduction

Next-generation sequencing technologies with higher sensitivity combined with appropriate bioinformatics analysis and artificial intelligence algorithms have significantly improved our understanding of the mutational landscape of human cancers [1,2]. The precise application of targeted therapies has become an increasing possibility over the last two decades as comprehensive genomic profiling (CGP) assays unravel an increasing number of mutations in targetable pathways. This ensures the important role of tumor genetic testing for the selection of effective therapeutics [3,4].

Assays on next-generation sequencing (NGS) technology, when developed with scientific precision, can highlight intricate genetic portraits of a patient’s tumor [5]. The comprehensive genomic profile obtained facilitates the discernment of clinically significant genetic anomalies that may be harnessed as potential therapeutic targets [6]. CGP assays can be applied to both tissue and liquid biopsy samples. A CGP thus enables the parallel detection of a wide array of genetic alterations, encompassing insertions, deletions, fusions, amplifications, rearrangements, and gene mutations [7,8].

CGP is an emerging need in the current cancer genomics landscape [9,10]. Unlike tumor-specific targeted gene panels, CGP covers key genes in totality, i.e., full exons and certain crucial intronic regions that become essential to assist in disease treatment, management, and monitoring beyond the established and emerging guidelines [11]. For advanced and complex tumors, CGP-based tests can render multiple actionable mutations for targeted therapeutics [12]. In addition, the cell -free or circulating tumor DNA (ctDNA), circulating tumor cells (CTCs), and single-cell genomic analysis are of increasing interest, making precision oncology the most promising tool for personalized medicine [13]. Several such actionable assays are approved for clinical practice, with multiple gene panels suggesting CGP [14,15].

The ever-evolving landscape of tumor biology, drug development, and immunotherapy is certainly driving toward the swift embrace of precision oncology [16,17,18].

NGS test validation is crucial and complex, involving multiple stages, such as preclinical trials, clinical trials, and regulatory approvals. Adequate sample selection is vital for robust validation, as it directly impacts clinical performance and the test’s purpose. Regulatory approval is essential to ensure reliability and compliance. Meeting predefined performance standards is key. Comprehensive validation, from sample preparation to reporting, ensures high-quality outcomes [19,20,21]. NGS test validation also requires the assessment of technical and computational aspects, as the amount of data output is huge [21,22]. MSI as a biomarker includes over hundreds and thousands of data points, the scoring of which requires the NGS tool to study the allelic makeup at all the considered MSI sites, later averaged as a single score. A threshold based on validation samples including patients is then used to stratify into responders and non-responders [20]. In addition to predicting immunotherapy response, high-frequency MSI (MSI-H) is also recognized as a potential marker for identifying and categorizing germline mutations in certain DNA mismatch repair (MMR) genes associated with Lynch syndrome [23,24,25]. Samples used for validation play a crucial role in establishing the detection sensitivity of the algorithm, consecutively upending necessary standards for reporting on a clinical backdrop. Thus, the objective of this study is to validate the NGS-based OncoIndx^®^ test with NGS standard references, clinical samples, and U.S. Food and Drug Administration (FDA)-approved cross-laboratory samples to determine its analytical performance and precision.

We show the prediction of optimal treatment strategies (precision therapies and immunotherapies), based on the identification of biomarkers in more than 4000 clinical samples (cohort-based publication underway) with proven and published links to approved therapies and precision medicine clinical trials (CTs) within a sizable population of advanced cancer patients. The assay includes 339 intronic regions, 15,719 exonic regions, 50 fusions, including 138 fusion partners, homologous recombination deficiency (HRD) based on over 50 homologous recombination repair (HRR) markers, genome-wide loss of heterozygosity (LOH), large-scale transitions (LSTs), and the telomeric allelic imbalance (TAI) of the 1080 genes covered in the panel. In addition, the assay covers 36 pharmacogenomic markers compatible with both liquid and tissue biopsies for a range of solid tumors. It has the capability to detect single nucleotide variants (SNVs), CNAs, structural variants, microsatellite instability (MSI), and tumor mutational burden (TMB). With the average coverage depths of 2000× for tissue and 10,000× for blood, immunotherapy biomarkers, including microsatellite instability (MSI), and tumor mutational burden (TMB) are detected and validated in this study with NGS reference samples, clinical samples, and cross-laboratory samples.

The OncoIndx assay has been useful to determine optimal treatment strategies (precision therapies and immunotherapies) based on the identification of genomic biomarkers in more than 4000 clinical samples (cohort-based publication underway) with proven and published links to approved therapies and precision medicine clinical trials (CTs).

## 2. Materials and Methods

### 2.1. Sample Collection and Targeted Exon Sequencing

**Figure 1:**
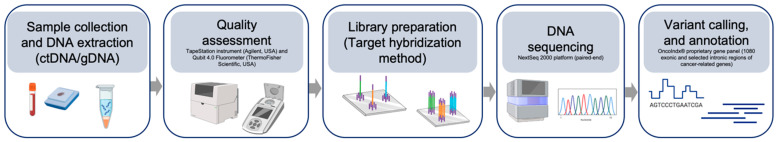
**Illustration of OncoIndx^®^ workflow.** The Figure presents the detailed workflow of OncoIndx NGS assay from sample collection, DNA extraction, DNA sequencing, variant calling, variant annotation, and data analysis (Created with BioRender.com).

A total of 63 patient samples, including industry reference standards (cfDNA and gDNA), clinical samples (blood and formalin-fixed paraffin embedded tissue (FFPE) samples), and cross-laboratory samples were utilized in this study. The ctDNA or FFPE DNA were extracted using commercially available DNA extraction kits (QIAamp minELute ccfDNA DNA kit, Qiagen, Hilden, Germany). The extracted DNA was subjected to a quality check (≥80% ccfDNA content) on a TapeStation 4200 instrument (Agilent Technologies, Santa Clara, CA, USA), and the concentration was determined on a Qubit 4.0 fluorometer (ThermoFisher Scientific, Waltham, MA, USA). Quality assessment was followed by the preparation of Illumina-compatible libraries with OncoIndx panel using target hybridization method. DNA libraries were then sequenced on the NextSeq 2000 (Illumina, San Diego, CA, USA) platform in a paired-end fashion (150 × 2). Variant calling and annotation was then performed using the indigenously developed iCare platform. An illustration of the NGS workflow is presented in Figure 1.

### 2.2. Bioinformatic Data Processing

Post-NGS analysis involves trimming the adapter and barcode sequences from the raw FastQ files upon thorough quality assessment (QA tests). Trimmed sequences were then aligned with the Genome Reference Consortium Human Build 38 (GRCh38) reference genome. Further, variant calling involved using the proprietary iCare^TM^ software platform (https://www.icaretm.com/, accessed on 1 July 2024).

### 2.3. Variant Prioritization and Interpretation

Filtered genomic variants from the variant calling pipeline were prioritized using cancer databases like ClinVar–NCBI (https://www.ncbi.nlm.nih.gov/clinvar/, accessed on 1 July 2024), as well as our proprietary curated database on the iCare^TM^ software platform. Additionally, in silico prediction tools, including SIFT, POLYPHEN, etc., were added for prioritizing variants. Variants were then interpreted based on tier level categorization from tier I, tier II, and tier III levels (as per the Association for Molecular Pathology, AMP), which indicates the variants with strong clinical significance (level A and B evidence), variants with potential clinical significance (level C or D evidence), and variants with unknown clinical significance, respectively [26]. Finally, molecular therapies were recommended along with published, recruiting, and potential clinical trials.

### 2.4. Validating Test Outcomes

#### 2.4.1. Level 1: Reference Standards

The first level of extensive validation of the OncoIndx^®^ comprehensive panel was performed using 43 NGS standard reference materials from Seraseq™ (Milford, MA, USA) with known true mutations, including SNV, small InDels, CNA, and fusions. Reference standard samples at tumor fractions 5%, 4%, 3%, 2%, 1%, and 0.1% were assessed to determine the detection efficiency and limit of detection (LOD). The reproducibility of variant detection across different batches of samples was also estimated for OncoIndx^®^. Sample analyses were repeated for 5 sequencing batches whose outcomes were then investigated.

#### 2.4.2. Level 2: Clinical Samples

As level 2 performance validation, 14 clinical samples sequenced by the OncoIndx^®^ panel were analyzed. The concordance between the OncoIndx^®^ test findings and clinically established hotspot findings were established for each clinical sample.

#### 2.4.3. Level 3: Orthogonal Validation

Finally, the OncoIndx^®^ test detection efficiency of immuno–oncology biomarkers, the tumor mutation burden (TMB), and microsatellite instability (MSI) were validated against 6 cross-laboratory samples whose results were produced by accredited reference laboratory tests.

## 3. Results and Discussion

### 3.1. OncoIndx^®^ Detected Genomic Alterations from NGS Standard Reference Samples with High Concordance and Analytical Precision

OncoIndx^®^ was tested to detect SNVs, INDELs, CNAs, fusions, and biomarkers like TMB, MSI, HRD from industry NGS reference samples that were pre-synthesized with variants. The outcomes are discussed in detail in the following sections.

#### 3.1.1. Single Nucleotide Variants and INDELs

The clinical implementation of genomic tests requires high analytical precision. Several parameters, including accuracy, sensitivity, and specificity, stand vital for such investigations. Thus, the OncoIndx^®^ variant calling pipeline (iCare’s VCP)© was validated using 43 NGS reference samples with variant distributions at a 5%, 1%, and 0.1% frequency. From the test outcomes, variant detection was most sensitive and accurate at 5% variant allele frequency (VAF) was reported in Table 1. CNAs were detected with 100% accuracy, sensitivity, and specificity, with no false positive and/or false negative occurrences. From a total of 264 SNVs detected by OncoIndx^®^, 156 were true positives and 108 rendered true negative outcomes with excellent concordance against reference standards. The highest positive and negative predictive values (NPVs and PPVs) obtained from the OncoIndx^®^ assay were 100% for SNVs. A maximum accuracy of 97.40% was obtained for small INDELs with 100% specificity and 95.60% sensitivity. In addition, fusions were detected with a high accuracy of 98.48%. It also yielded 100% specificity and a sensitivity of 97.44%. The OncoIndx^®^ assay pipeline yielded no false positive hits, even at 1% VAF. This highlights the reliability and sensitivity of the assay to exhibit acceptable performance for variant calling even at 1% VAF. The analytical parameters at a 1% and 0.1% VAF are presented in Tables S1 and S2.

#### 3.1.2. Copy Number Alterations and Fusions

**Figure 2:**
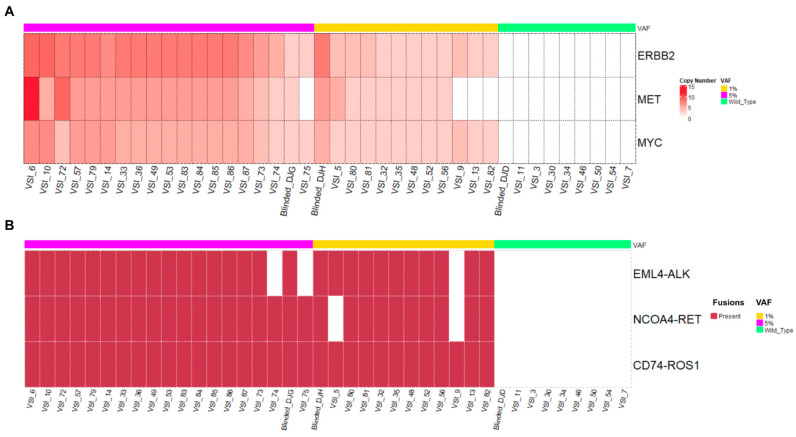
**Distribution of copy number alterations and fusions detected by OncoIndx.** (**A**) From the total number of samples in the cohort of 5% VAF, this Figure indicates 39 true positive copy number alterations with 0 false positives detected across the reference sample validation cohort. (**B**) This Figure indicates the detection of fusions with 38 true positives and 1 false positive. In both A and B, no CNAs/fusions were detected in wild-type controls.

The detection of CNAs and fusions can be challenging due to inconsistencies of rearranged chromosomal regions, breakpoints, read lengths, etc. SVs such as fusions may be equally important in solid tumors as they are in hematological cancers [27]. In OncoIndx^®^, the detection of CNAs and fusion variants were validated using customized reference materials at VAF from 5% to 1% and 0.1%. The validation set was mainly focused on three genes for CNAs, namely ERBB2, MET, and MYC, and three fusions, namely EML4-ALK, NCOA4-RET, and CD74-ROS1. CNAs were detected with a highest PPV, NPV, specificity, and accuracy of 100%. In addition, no false positives and false negatives were detected from the OncoIndx^®^ assay for CNAs at 5% VAF, whose distributions are shown in Figure 2A. A total of 100% of patients with ERBB2 and MYC amplification were detected at a VAF as low as 0.1% (Figure 2A). Next, for fusions, a detection accuracy of 98.48% and a PPV and specificity of 100% were obtained with no false positive detection. At all three dilutions of VAF (5%, 1%, and 0.1%), fusions were detected at a specificity of 100%. NCOA4-RET and CD74-ROS1 fusions were predominantly detected at 0.1%VAF, indicating the sensitivity of the OncoIndx^®^ assay at a low VAF of 0.1% (Figure 2B and Figure S1).

#### 3.1.3. Limit of Detection of OncoIndx^®^

**Figure 3:**
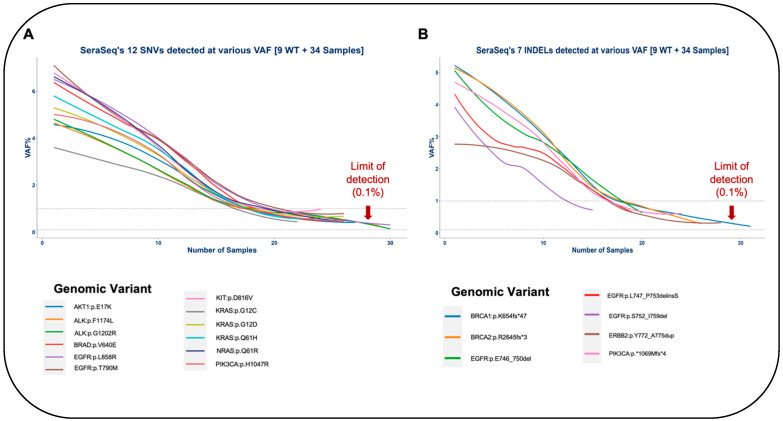
Limit of detection of OncoIndx^®^ test. (**A**,**B**) Detection limit observed at 0.1% VAF for majority of SNVs and INDELs pre-established by standard reference samples tested by OncoIndx^®^ assay (shown by arrows).

For identifying the limit of detection (LOD) of OncoIndx^®^ assays, serial dilutions of standard reference Seraseq™ samples were performed from 0.1% to 1% VAF. Gene variants including AKT1:p.E17K, EGFR:p.L858R, EGFR:p.E746_A750del, and ERBB2:p.Y772_A775dup were detected in each of the diluted samples. Expected vs. observed VAF% were visualized for all variants to further determine LOD. A total of 83.33% (n = 5) of SNVs could be detected at a VAF less than 1%, namely, EGFR (p.T790M), NRAS (p.Q61R), PIK3CA (p.H1047R), KRAS (p.G12D), and ALK (p.G1202R), respectively (Figure 3A). Similarly, for small InDels, 60% (n = 3) of the variants could be detected at a VAF less than 1%, namely, BRCA1 (p.K654fs*47), BRCA2 (p.R2645fs*3), and PIK3CA (p.*1069Mfs*4) (Figure 3B). The observed % VAF thus denotes the LOD of OncoIndx^®^ assays, which was determined for both SNVs and INDELs. Thus, the overall sensitivity of OncoIndx^®^ appears at 0.1% from the LOD values, as indicated by the arrows in Figure 3A,B. A list of SNVs and INDELs validated from the industrial samples is presented in Table 2.

### 3.2. High Concordance of Genomic Alterations Obtained from Clinical Samples

A clinical sample cohort with 14 samples was tested across genomic alterations for five hotspot genes, namely EGFR, ALK, KRAS, PIK3CA, and BRCA2. The OncoIndx^®^ assay showed 100% concordance for alterations detected in the EGFR, ALK, KRAS, and BRCA2 genes, and 50% concordance for PIK3CA alterations (Table 3). In ALK-positive samples, the OncoIndx^®^ assay efficiently determined ALK fusion partners beyond EML4, including NPM1. In addition, we predict that the 50% concordance obtained for PIK3CA alteration in one clinical sample may most likely be due to tumor heterogeneity presented by testing different sample types, i.e., a former clinical finding being in the FFPE sample and the OncoIndx^®^ assay in the ctDNA from a blood sample.

Tumor heterogeneity is a major roadblock in effective treatment decision-making, which is also one of the main challenges of FFPE DNA testing. Thus, ctDNA-based genomic detection may offer benefits in these cases by analyzing the totality of a tumor rather than individual sections like the former testing method. Thus, OncoIndx^®^ may be a beneficial complementary tool in such scenarios. In addition to its concordance among genomic alterations, the OncoIndx^®^ assay was also validated against important biomarkers, including MSI, TMB, LOH, LST, and TAI, whose outcomes are detailed in the next section.

### 3.3. Validation of Biomarker Signatures against Reference Laboratories: Microsatellite Instability and Tumor Mutation Burden

In addition to detecting genomic variants, the OncoIndx^®^ test identifies the status of biomarkers to predict immunotherapy response in patients. From the blood samples collected, MSI and TMB were detected. MSI in tumors has been studied to promote “immune-hot” conditions [28,29,30]. Thus, the presence of high MSI favors tumor immune responses. To validate the reproducibility of MSI detected from OncoIndx^®^, two MSI detection tools were utilized against the outcomes of clinically stratified reference laboratory samples. From our pipeline, MSI was detected with an accuracy of 95%, a sensitivity of 90%, and a specificity of 100%. Overall, the PPV was identified to be 100% and the NPV was 90.91% (Table 4).

**Figure 4:**
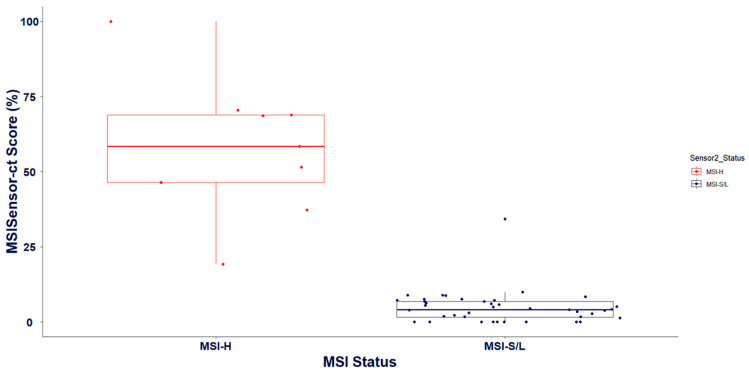
Validation of MSI status from cross-laboratory documentations across MSI prediction in OncoIndx^®^.

All high-MSI and low-MSI samples were appropriately predicted, with one misclassified sample whose value was in the intermediate range (Figure 4). Intermediate MSI values are challenging as they are neither high nor low and are missed in many classifier tools. Thus, more optimizations are necessary and are constantly underway in OncoIndx^®^ for better accuracy.

The summarized outcomes from the reference laboratory validation of MSI against the Food and Drug Administration (FDA)-approved companion diagnostic tests are presented in Table 5. These outcomes suggest the high sensitivity and specificity of the OncoIndx^®^ assay in detecting MSI from NGS.

With rapidly emerging immuno–oncological biomarkers, MSI stands important for immunotherapy selection, and a highly sensitive assay like OncoIndx^®^ can improve the responsive standards of immunotherapy in cancer [31]. The MSI threshold criteria utilized for our CGP test are presented in Table 6.

Likewise, TMB predictions from the OncoIndx^®^ assay were cross-validated with reference standards like Seraseq^™^ and College of American Pathologists (CAP)-accredited genomic DNA lab reference samples. Reference laboratory validations against the FDA-approved tests were also performed using OncoIndx^®^ CGP (Table 7 and Table 8).

The TMB thresholds utilized in the CGP assay are presented in Table 9. The outcomes have been categorized as low and high TMB scores, which are predictive of immunotherapy benefits.

**Figure 5:**
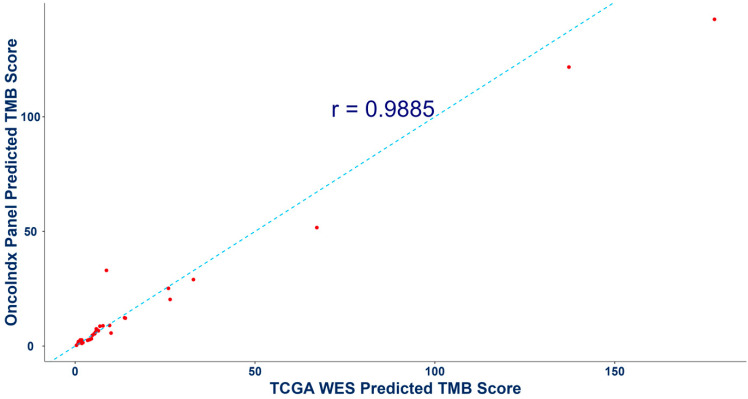
**Pearson correlation outcomes for tumor mutation burden from whole-exome sequencing and OncoIndx gene panel.** This Figure shows a high correlation between the predicted TMB scores of whole-exome sequencing (WES) and the targeted comprehensive gene panel from OncoIndx.

In addition, the predicted TMB values from OncoIndx^®^ panel were also validated by comparing with whole-exome sequencing (WES) data from the Cancer Genome Atlas Program (TCGA) database. A Pearson correlation coefficient of r = 0.9885 was obtained (Figure 5). Overall, these outcomes indicate concordance between the OncoIndx^®^ CGP and established FDA-approved companion diagnostic tests.

The authors would also like to acknowledge few drawbacks of the current study as will be taken up in the future, including the need for more validation samples, especially at the 0.1% VAF dilution, and also increased cross-laboratory validation to improve orthogonal validation.

## 4. Conclusions

In conclusion, the performance of the OncoIndx^®^ assay for SNVs, small INDELs, CNAs, and fusions was optimally validated against NGS reference standards for analytical validation, patient samples for clinical validation, and cross-laboratory testing for orthogonal validation. The outcomes prove the OncoIndx^®^ assay to be an effective tool for NGS analysis. In addition, the test performance was evaluated at multiple VAFs, and the results show that OncoIndx^®^ can detect variants and perform optimally at VAFs as low as 0.1%.

Overall, OncoIndx^®^ comprises markers for precisely targeted therapies, immunotherapy, and selected chemotherapies, which are all designed to interrupt oncogenic processes and regulate molecular pathways that either drive the disease or imbibe resistance. Integrating the comprehensive capabilities of OncoIndx^®^ with the rigorous clinical validation required for NGS tests, we ensure a deep understanding of cancer genetics as well as the highest standards of accuracy and reliability in clinical practice.

## Figures and Tables

**Table 1:** Outcomes of statistical analysis obtained from OncoIndx^®^ comprehensive genomic panel at 5% variant allele frequency.

Alteration Type	Total Number of Alterations	True Positives	False Positives	True Negatives	False Negatives	* PPV	* NPV	Accuracy	Specificity	Sensitivity
SNVs	264	156	0	108	0	100	100	100	100	100
Small INDELs	154	87	0	63	4	100	94.03	97.40	100	95.60
CNA	66	39	0	27	0	100	100	100	100	100
Fusions	66	38	0	27	1	100	96.43	98.48	100	97.44

* NPV: Negative predictive value. PPV: Positive predictive value.

**Table 2:** This Table presents the list of SNVs and INDELs detected and validated from the industrial samples.

List of SNVs and INDELs Validated from the Industrial Samples	
AKT1:p.E17K	EGFR:p.T790M
ALK:p.F1174L	ERBB2:p.Y772_A775dup
ALK:p.G1202R	KIT:p.D816V
BRAF:p.V640E	KRAS:p.G12C
BRCA1:p.K654fs*47	KRAS:p.G12D
BRCA2:p.R2645fs*3	KRAS:p.Q61H
EGFR:p.E746_A750del	KRAS:p.Q61R
EGFR:p.L747_P753delinsS	NRAS:p.Q61R
EGFR:p.L858R	PIK3CA:p.*1069Mfs*4
EGFR:p.S752_I759del	PIK3CA:p.H1047R

**Table 3:** This Table shows the concordance levels of genomic alterations in clinical samples as detected by the OncoIndx^®^ assay.

S. No.	Genes	Concordant Genomic Findings from OncoIndx^®^ Assay	Concordance Levels Obtained from OncoIndx^®^ Assay
1	EGFR	L858R E746_A750del L747_S752del	100%
2	ALK	NPM1-ALK ALK-EML4 Fusion G1202R G1269A	100%
3	KRAS	A146T	100%
4	PIK3CA	H1047R	50%
5	BRCA2	S636*	100%

**Table 4:** This Table presents the analytical parameters of MSI detection by OncoIndx^®^ test.

Statistics of MSI Detection in OncoIndx^®^ (Percentage %)	
Positive Predictive Value (PPV)	100
Negative Predictive Value (PPV)	90.91
Sensitivity	90
Specificity	100
Accuracy	95

**Table 5:** OncoIndx^®^ test prediction of MSI status validated against FDA-approved test outcomes.

Sample Type	FDA-Approved Test Prediction	OncoIndx^®^ Test Prediction
Blood	MSS	3.2 (MSI-low)
Blood	MSS	1.55 (MSI-low)
Blood	MSS	0.79 (MSI-low)
Blood	MSS	3.07 (MSI-low)
Blood	MSS	3.61 (MSI-low)

MSS: Microsatellite stability.

**Table 6:** MSI thresholds of high, intermediate, low, and stable scores utilized for various sample types in OncoIndx^®^ test.

Biomarker/s	Outcome	Blood/Pleural Effusion	FFPE/RNALater
MSI	MSI-H	≥20	≥20
	MSI-I	≥10	≥10
	MSI-L	<10	<10
	MSI-S	0	0

**Table 7:** TMB validation performed against NGS reference standards.

Sample	Sample Type	True Prediction	OncoIndx^®^ Test Prediction
Control	Healthy control	Negative control	1.5
	Healthy control	Negative control	1.5
	Healthy control	Negative control	1.5
	Healthy control	Negative control	2.5
	Healthy control	Negative control	1.67
	Healthy control	Negative control	0
	Healthy control	Negative control	0
	Healthy control	Negative control	0.5
SeraSeq^TM^ reference samples	TMB Mix Score 7 (0%)	5.8–9.2	8.67
	TMB Mix Score 7 (0.5%)	10.5–15.7 (d = 3.5–7.7)	10.83
	TMB Mix Score 7 (2%)	16.6–19.2 (d = 3.5–7.7)	6.67
	TMB Mix Score 20 (0%)	6.1–8.9	6.5
	TMB Mix Score 20 (0.5%)	23.7–28.3 (d = 15.8–21.2)	8.17
	TMB Mix Score 20 (2%)	34.6–36.6 (d = 26.4–29.8)	5.83
CAP gDNA samples	gDNA	9	11.5
	gDNA	26	19.5
	gDNA	9	9.83
	gDNA	26	5.67

**Table 8:** Cross-laboratory validation of TMB with OncoIndx^®^ test predictions.

Sample Type	FDA-Approved Test Prediction	OncoIndx^®^ Test Prediction
Blood	1	3.2
Blood	7.26	1.55
Blood	6.7	0.79
Blood	3	3.07
Blood	4	3.61
Blood	4.77	3.167

**Table 9:** TMB thresholds of OncoIndx^®^ test.

Biomarker/s	Outcomes	Blood/Pleural Effusion	FFPE/RNALater
TMB	TMB-H	≥10	≥10
	TMB-L	<10	<10

## Data Availability

Data are not publicly available due to privacy restrictions. The data that support the findings of this study are available from the corresponding authors, G.S. and J.K., upon reasonable request.

## Supplementary Materials

The following supporting information can be downloaded at: https://www.mdpi.com/article/10.3390/cancers16193415/s1, Table S1: Outcomes of statistical analysis l at 1% variant allele frequency; Table S2: Outcomes of statistical analysis at 0.1% variant allele frequency; Figure S1: Distribution of (**A**) copy number alterations and (**B**) Fusions detected by OncoIndx at 5%, 1%, and 0.1% VAF against Wild-type controls.
