# Supplementary material for: Clinical and Technical Validation of OncoIndx® Assay—A Comprehensive Genome Profiling Assay for Pan-Cancer Investigations
Source: Cancers (Basel). 2024 Oct 8;16(19):3415. doi: 10.3390/cancers16193415 (PMC11475161; doi:10.3390/cancers16193415)
Supplement: Supplementary file 1 [file cancers-16-03415-s001.zip › cancers-3151648-supplementary.pdf]

Table S1. Outcomes of statistical analysis I at 1% variant allele frequency.

Alteration type	Total number of alterations	True positives	False positives	True negatives	False negatives	*PPV	*NPV	Accuracy	Specificity	Sensitivity
SNVs	276	145	0	108	23	100	82.44	91.67	100	86.31
Small INDELs	161	67	0	63	31	100	67.02	80.75	100	68.37
CNA	75	33	0	27	15	100	64.29	80.00	100	68.75
Fusions	75	33	0	27	15	100	64.29	80.00	100	68.75

Table S2. Outcomes of statistical analysis at 0.1% variant allele frequency.

Alteration type	Total number of alterations	True positives	False positives	True negatives	False negatives	*PPV	*NPV	Accuracy	Specificity	Sensitivity
SNVs	192	16	0	108	68	100	61.36	64.58	100	19.05
Small INDELs	112	8	0	63	41	100	60.58	63.4	100	16.33
CNA	48	10	0	27	11	100	71.05	77.08	100	47.62
Fusions	48	3	0	27	18	100	60	62.5	100	14.29

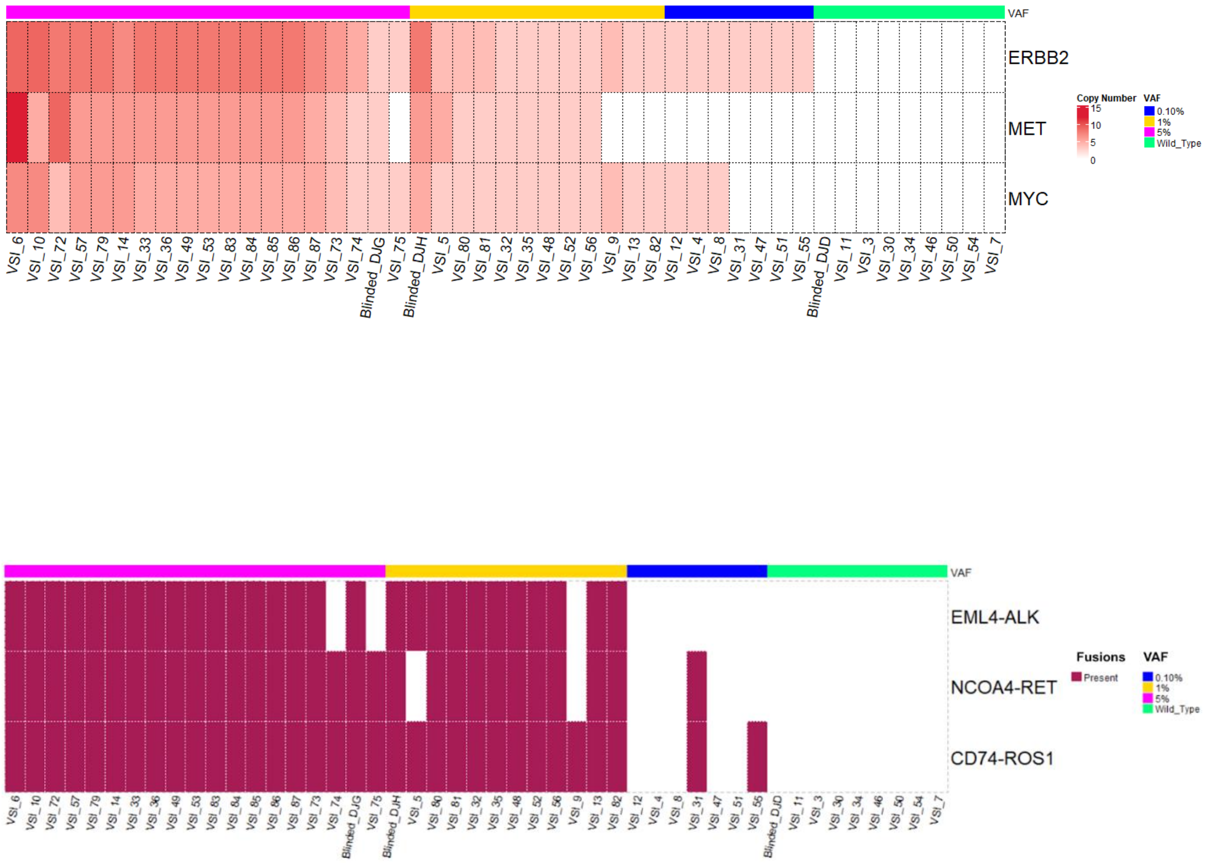

Figure S1. Distribution of **(A)** copy number alterations and **(B)** Fusions detected by OncoIndx at 5%, 1%, and 0.1% VAF against Wild-type controls.
